# Supplementary material for: Using population selection and sequencing to characterize natural variation of starvation resistance in Caenorhabditis elegans
Source: eLife. 2022 Jun 21;11:e80204. doi: 10.7554/eLife.80204 (PMC9262388; doi:10.7554/eLife.80204)
Supplement: Transparent reporting form [file elife-80204-transrepform1.docx]

***eLife’s* transparent reporting form**

We encourage authors to provide detailed information *within their submission* to facilitate the interpretation and replication of experiments. Authors can upload supporting documentation to indicate the use of appropriate reporting guidelines for health-related research (see [EQUATOR Network](http://www.equator-network.org/%20)), life science research (see the [BioSharing Information Resource](https://biosharing.org/)), or the [ARRIVE guidelines](http://www.plosbiology.org/article/info:doi/10.1371/journal.pbio.1000412) for reporting work involving animal research. Where applicable, authors should refer to any relevant reporting standards documents in this form.

If you have any questions, please consult our Journal Policies and/or contact us: [editorial@elifesciences.org](mailto:editorial@elifesciences.org).

**Sample-size estimation**

- You should state whether an appropriate sample size was computed when the study was being designed
- You should state the statistical method of sample size computation and any required assumptions
- If no explicit power analysis was used, you should describe how you decided what sample (replicate) size (number) to use

Please outline where this information can be found within the submission (e.g., sections or figure legends), or explain why this information doesn’t apply to your submission:

Parameters for MIP-seq were determined by the pilot experiments in Figure 1. Power analyses were performed for starvation survival to determine effect sizes for negative results (described in the Methods section), but explicit power analyses were not performed for starvation recovery and early fecundity assays given significant results. Based on our extensive prior experience with these assays, we know that 3-5 biological replicates is sufficient to detect relatively small differences for these assays.

**Replicates**

- You should report how often each experiment was performed
- You should include a definition of biological versus technical replication
- The data obtained should be provided and sufficient information should be provided to indicate the number of independent biological and/or technical replicates
- If you encountered any outliers, you should describe how these were handled
- Criteria for exclusion/inclusion of data should be clearly stated
- High-throughput sequence data should be uploaded before submission, with a private link for reviewers provided (these are available from both GEO and ArrayExpress)

Please outline where this information can be found within the submission (e.g., sections or figure legends), or explain why this information doesn’t apply to your submission:

The number of biological replicates performed is indicated in figure legends. Each biological replicate involves repeating the experiment again at a different time, starting with setting up worms on new plates (described in ‘MIP-seq experimental set-up’ in the methods section). High-throughput sequencing data has been deposited on NCBI SRA (for MIP-seq data). Accession numbers were provided in the submission.

**Statistical reporting**

- Statistical analysis methods should be described and justified
- Raw data should be presented in figures whenever informative to do so (typically when N per group is less than 10)
- For each experiment, you should identify the statistical tests used, exact values of N, definitions of center, methods of multiple test correction, and dispersion and precision measures (e.g., mean, median, SD, SEM, confidence intervals; and, for the major substantive results, a measure of effect size (e.g., Pearson's r, Cohen's d)
- Report exact p-values wherever possible alongside the summary statistics and 95% confidence intervals. These should be reported for all key questions and not only when the p-value is less than 0.05.

Please outline where this information can be found within the submission (e.g., sections or figure legends), or explain why this information doesn’t apply to your submission:

(For large datasets, or papers with a very large number of statistical tests, you may upload a single table file with tests, Ns, etc., with reference to sections in the manuscript.)

The statistical tests used are indicated in the figure legends and further details of each test are described in the methods section for the corresponding assay. All individual data points are plotted whenever possible, effect sizes are indicated on the figures for worm length assays, and R^2 values are indicated for correlation analysis. All raw data used to generate figures and perform statistics is available in source data, supplementary files, or NCBI databases.

**Group allocation**

- Indicate how samples were allocated into experimental groups (in the case of clinical studies, please specify allocation to treatment method); if randomization was used, please also state if restricted randomization was applied
- Indicate if masking was used during group allocation, data collection and/or data analysis

Please outline where this information can be found within the submission (e.g., sections or figure legends), or explain why this information doesn’t apply to your submission:

Samples were allocated into experimental groups based on genetic background, as indicated throughout the manuscript, as the goal was to determine whether genetic background affected starvation resistance. Masking was not used.

**Additional data files (“source data”)**

- We encourage you to upload relevant additional data files, such as numerical data that are represented as a graph in a figure, or as a summary table
- Where provided, these should be in the most useful format, and they can be uploaded as “Source data” files linked to a main figure or table
- Include model definition files including the full list of parameters used
- Include code used for data analysis (e.g., R, MatLab)
- Avoid stating that data files are “available upon request”

Raw sequencing data is available in the appropriate databases (NCBI SRA). Processed sequencing data, and analysis of MIP-seq, GWAS, and RNA-seq data is available in supplementary data files. Source data for phenotypic assays (including starvation survival, worm length, and nuclear localization assays) is available in source data files. Scripts to process MIP-seq data are available on github as indicated in the manuscript.

Please indicate the figures or tables for which source data files have been provided:
